# Endocrine Challenges in Myoclonic Epilepsy With Ragged Red Fibers Syndrome: A Case Report

**DOI:** 10.7759/cureus.51114

**Published:** 2023-12-26

**Authors:** Henrique Carmona Alexandrino, Marta A Ferreira, Diogo Ramalho, Nuno R Jesus, Maria J Oliveira

**Affiliations:** 1 Department of Endocrinology, Centro Hospitalar de Vila Nova de Gaia/Espinho, EPE, Vila Nova de Gaia, PRT; 2 Cancer Signalling & Metabolism, Instituto de Investigação e Inovação em Saúde da Universidade do Porto (i3S), Porto, PRT

**Keywords:** disease complexity, comprehensive patient care, endocrine dysfunction, multidisciplinary approach, mitochondrial-endocrine interactions, clinical case report, multisystem disorders, endocrinopathies, merrf syndrome, mitochondrial dysfunction

## Abstract

Myoclonic epilepsy with ragged red fibers (MERRF) syndrome is a primary mitochondrial disorder characterized by myoclonus, epilepsy, ataxia, and muscle fiber abnormalities. While traditionally associated with neurological features, MERRF's multisystem nature extends to endocrine dysfunction, including diabetes mellitus, thyroid disorders, and adrenal abnormalities.

This case report explores the multifaceted nature of MERRF syndrome by presenting the clinical journey of a 70-year-old woman who sought care at the endocrinology clinic due to coexisting Addison's disease and diabetes mellitus, marked by recurrent hypoglycemia and suboptimal metabolic control. Over time, she developed a history of myoclonic epilepsy, effectively managed with lamotrigine, along with mild sensory axonal polyneuropathy and ataxia. The patient was diagnosed with MERRF syndrome following her son's diagnosis, which had a severe form. This case underscores the intricate interplay between mitochondrial dysfunction and endocrine manifestations in MERRF syndrome, highlighting the importance of a comprehensive and multidisciplinary approach to patient care.

MERRF syndrome's array of endocrine manifestations substantially impacts patients' quality of life and morbidity. A comprehensive approach, uniting endocrinologists, neurologists, geneticists, and other specialists, is essential for effective patient care. Further research is warranted to unravel the complex mitochondrial-endocrine interactions in MERRF syndrome, offering potential insights for improved management.

## Introduction

Primary mitochondrial diseases encompass a heterogeneous group of multisystem disorders arising from pathogenic genetic variants in mitochondrial DNA (mtDNA), resulting in mitochondrial respiratory chain dysfunction and impaired energy production [1]. 

MERRF (myoclonic epilepsy with ragged red fibers) is one such mitochondrial disorder characterized by myoclonus, epilepsy, ataxia, and muscle fiber abnormalities. The most prevalent cause is the m.8344A>G variant, which accounts for 80% of cases, with additional variants contributing 10%. Approximately 90% of MERRF cases involve maternally inherited mtDNA variants [2]. 

While neuromuscular symptoms often dominate, it is essential to recognize MERRF's multisystem nature, potentially including endocrine dysfunction. Endocrinopathies, such as diabetes, thyroid disorders, parathyroid dysfunction, and adrenal abnormalities, can either co-occur with mitochondrial disease or be part of the disease spectrum itself. Therefore, a meticulous evaluation and ongoing endocrine monitoring are crucial components of patient care [3].

In this case report, we aim to illustrate the significant role of endocrinological evaluation in elucidating and managing the complexities of MERRF syndrome. The case presented herein serves as an exemplar of the intricate interplay between mitochondrial dysfunction and endocrine manifestations in this syndrome.

## Case presentation

A 70-year-old woman was referred to our outpatient endocrinology clinic due to the management of primary adrenal insufficiency (Addison's disease) and diabetes mellitus (DM) with recurrent hypoglycemia and suboptimal metabolic control.

Medical history

The patient had a significant past medical history of myoclonic epilepsy, part of the MERRF syndrome spectrum, as well as chronic kidney disease 3A (no proteinuria), chronic atrophic gastritis (positive anti-parietal cell antibody), dyslipidemia, and benign paroxysmal positional vertigo. She was on multiple medications, including hydrocortisone 10mg (one in the morning, half at lunch, and half in the afternoon), fludrocortisone 150mcg OD, insulin degludec 18 units, pioglitazone 30mg OD, dapagliflozin 10mg, alprazolam 1mg BID, clonazepam 2mg BID, duloxetine 60mg OD, lamotrigine 100mg TID, and atorvastatin 40mg OD.

Diagnosis and management

The patient’s journey began 30 years earlier, when she was first diagnosed with primary adrenal insufficiency during an acute adrenal crisis. Laboratory investigations conducted at that time unveiled notably elevated ACTH levels (>1000 pg/mL) and diminished cortisol levels (<3 mcg/dL), with no documented measurements of specific antibodies (such as 21-hydroxylase antibodies) or recorded results for aldosterone and renin levels. Additionally, her thyroid function exhibited a TSH at the lower end of normal for quite some time of 0.33 uUI/mL (0.27 to 4.2) with normal free T4 and T3 levels. Thyroid antibodies (anti-TPO, anti-Tg, and TSH receptor) were always negative, and thyroid ultrasound findings were unremarkable. Ten years later, the patient was diagnosed with DM during routine follow-up, with a fasting blood sugar level consistently >126 mg/dL, initially categorized as type 2 DM. There were no reports of weight loss, polyuria, polydipsia, or neurosensory hearing loss documented at that time. DM was primarily managed with oral antidiabetic medications, including metformin, pioglitazone, gliclazide, and sitagliptin. However, her glycemic control remained suboptimal, with HbA1c levels ranging from 8%-9% (10.2 to 11.8 mmol/L). Over the years, she developed end-organ complications, including non-proliferative diabetic retinopathy and mild distal peripheral neuropathy. The patient experienced recurrent, unpredictable hypoglycemic episodes coupled with periods of hyperglycemia, often exceeding 300 mg/dL. In the past five years, she has been initiated on insulin therapy, starting with glargine and subsequently switching to degludec, with frequent dose adjustments. Approximately three years ago, metformin was discontinued due to an episode of hyperlactacidemia (>6 mmol/L) that occurred during an acute febrile illness.

Neurological presentation and family history

Her myoclonic epilepsy was diagnosed 15 years prior and effectively treated with lamotrigine 100mg tid. Afterwards, she developed, over the course of years, mild sensory axonal polyneuropathy and ataxia. Unfortunately, the original MRI records are unavailable as they were paper-based and have since been lost. However, the available description documented mild cerebellar and cerebral atrophy, with no other notable changes observed. Her last electroencephalogram showed low-amplitude spikes, slow-spike waves at the posterior vertex, and generalized slow-spike waves. The MERRF diagnosis was made after her son was diagnosed with a severe form of the m.8344A>G variant in MT-TK. The patient has a mutation load of 32% in her mitochondrial DNA. Her last seizure was 10 years ago, but along the way, our patient somehow lost follow-up on her neurology appointment. 

The patient's family history revealed a significant occurrence of MERRF syndrome, linked to the m.8344A>G variant in the MT-TK gene. This genetic variant was identified not only in the patient but also in her two sons, her sister, and her nephew. The clinical presentation of MERRF syndrome in her family members displayed a wide range of severity. Notably, the patient's daughter exhibited a relatively mild form of the disease, with a mutation load of 32% in her mitochondrial DNA. In stark contrast, her son confronted a much more severe manifestation, featuring a mutation load of 92% in his mitochondrial DNA. Tragically, her son's condition led to his untimely demise at the age of 46. The primary cause of his passing was the involvement of respiratory muscles due to the disease, ultimately resulting in respiratory failure followed by infection.

Challenges in management

Returning to the present, the patient sought medical attention following an acute febrile illness, prompting a comprehensive evaluation of her fluctuating diabetes, characterized by frequent hypoglycemic episodes and hyperglycemia. Upon a meticulous review of her medical history and medication regimen, we made several therapeutic adjustments. Specifically, we transitioned her from insulin degludec to insulin-neutral protamine Hagedorn (NPH), administering 16 units once daily in the early morning while discontinuing pioglitazone (which should be avoided in mitochondrial diseases). We believe that insulin NPH would be a better option for this patient due to its duration of action (approximately 12-16 hours), which would prevent morning hypoglycemia and help lower her postprandial hyperglycemias. Another reason why we chose this treatment instead of a basal-bolus approach was due to the high burden of disease and the need for a simpler regimen.

Regarding her Addison's disease, we scheduled a group education session for patients with adrenal insufficiency and provided her with a medical alert card (European Emergency Card). Additionally, we initiated coenzyme-Q supplementation (100 mg thrice daily) and B vitamin complex to try to improve mitochondrial function. Recognizing the intricacies of her medical history, we promptly sought consultation with a neurologist to address her neurological symptoms and the complexities of MERRF syndrome.

Laboratory findings and treatment response and future management

Noteworthy laboratory findings included a slight normocytic anemia with iron deficiency and normal B12 and folic acid levels. Other laboratory results are a significantly elevated HbA1c of 10.8% a decreased C-peptide level of 0.6 ng/mL (1,1-4,4), with paired glucose of 226 mg/dL. The serum creatinine level was 1.04 mg/dL (0.7-1.3 mg/dL) with an estimated glomerular filtration rate of 57 mL/min/1.73 m² (2021 CKD-EPI creatinine formula) and no evidence of proteinuria. Additionally, her calcium phosphate metabolism, as well as her thyroid function tests, were also normal (Table 1).

**Table 1:** Endocrinological assessment of the patient ALT: Alanine Aminotransferase, AST: Aspartate Aminotransferase, HbA1c: Glycated Hemoglobin, PTH: Parathyroid Hormone, T3: Triiodothyronine, T4: Thyroxine, TIBC: Total Iron-Binding Capacity, TSH: Thyroid Stimulating Hormone.

	Result	Reference values
Hemoglobin	11.8	13.0-18.0 g/dL
Mean corpuscular volume	93	80-100 fL
Fasting Glucose	226	70-105 mg/dL
HbA1c	10.8	4-5.7 %
C-peptide	0.6	1.1-4.4 ng/mL
Creatinine	1.04	0.7-1.3 mg/dL
Urea	49	17-50 mg/dL
Sodium	140	135-145 mmol/L
Potassium	4.19	3.5-5 mmol/L
AST	21	4 - 27 U/L
ALT	19	4 – 34 U/L
Alkaline Phosphatase	52	35-104 U/L
Total calcium	8.4	8.2-8.8 mg/dL
Albumin	4.1	3.4-4.8 g/dL
PTH	52.3	15.0-65.0 pg/mL
Vitamin D	74	62.5-200 nmol/L
Folic Acid	5.2	4.6-18.7 ng/mL
Vitamin B12	709	197-771 pg/mL
Ferritin	14	13-400 ng/mL
Iron	35	37-145 ug/dL
TIBC	430	228-428 ug/dL
Transferrin	281	200-360 mg/dL
Transferrin saturation	10.1	15-55 %
TSH	2.67	0.27-4.2 uUI/mL
Free T4	1.35	0.93-1.70 ng/mL
Free T3	3.2	2.57-4.43 pg/mL
Urinary albumin excretion	27	< 30 mg/dL

Her DM showed improvement after titrating her insulin NPH dose to 22 units once daily, resulting in better glycemic control without reported hypoglycemia and occasional post-dinner hyperglycemia. Her family reports that she now feels better and experiences fewer hypoglycemic events, a crucial consideration given her coexisting Addison's disease and mitochondrial disorder. Regarding vitamin supplementation (coenzyme Q and B complex vitamins), although we do not expect any significant improvement in her condition, we hope to maintain her mitochondrial respiratory chain function and reduce the levels of reactive oxygen species arising from disrupted mitochondrial metabolism.

The patient has been regularly attending our follow-up appointments, allowing us to implement our management strategy effectively. Through routine endocrine evaluations every three to six months, encompassing thyroid and parathyroid function assessments, as well as scheduled cardiology evaluations involving electrocardiograms and echocardiograms, due to her heightened cardiovascular risk. Our approach also emphasizes patient education, particularly regarding the avoidance of mitochondrial toxins such as aminoglycoside antibiotics, linezolid, cigarettes, alcohol, and valproic acid. While the patient's adherence to these recommendations has been positive, the overall impact of our management strategy on her health has been encouraging. We've observed stabilization in certain aspects of her condition and witnessed a reduction in potential aggravating factors. However, we continue to adapt our strategy based on ongoing assessments and patient feedback to ensure the most effective management of her condition.

These findings and therapeutic adjustments underscore the multifaceted nature of managing a patient with MERRF syndrome and concurrent endocrine disorders, highlighting the need for a meticulous and collaborative approach.

## Discussion

In MERRF syndrome, traditionally characterized by hallmark features such as myoclonus, epilepsy, ataxia, and exercise intolerance, the association with diabetes mellitus and other endocrinopathies is not typically considered part of the clinical spectrum [4]. 

However, mitochondrial diseases, including MERRF syndrome, are recognized as multisystem disorders capable of affecting various organs. Organs with high energy demands, including the brain, eyes, heart, and skeletal muscles, are particularly susceptible to oxidative phosphorylation stress due to their reliance on efficient mitochondrial energy production [5]. The endocrine system is no exception, as all endocrine glands depend heavily on ATP for energy, making them vulnerable to mitochondrial dysfunction, which can impair hormone secretion and disrupt normal feedback regulation. The mechanisms underlying endocrine abnormalities in mitochondrial disorders are complex and not fully elucidated. There is emerging evidence to suggest that mitochondrial disorders may impact certain aspects of the immune system, potentially leading to increased production of reactive oxygen species, elevated mutation rates, and the initiation of apoptotic pathways within immune cells [3].

Mitochondrial genetics exhibits unique characteristics because, within a cell, mtDNA has a very high copy number (about hundreds or thousands of copies present). This gives rise to two distinctive conditions: homoplasmy, in which 100% of mtDNA copies in a cell share the same genetic type, and heteroplasmy, in which both mutant and wild type mtDNA coexist within the same cell. In the context of mitochondrial diseases, the majority of mtDNA mutations lead to a loss of function. To trigger organ dysfunction, a critical proportion of mtDNA in a specific tissue must bear these disease-causing mutations before any biochemical deficiency becomes apparent. However, it's important to note that the level of mutant mtDNA heteroplasmy doesn't always directly correspond to the clinical symptoms experienced by the patient [6,7].

Notably, data collected by the North American Mitochondrial Disease Consortium Patient Registry (last update of 2018), which systematically gathers information on commonly encountered endocrine conditions, has highlighted a disproportionate burden of endocrine disorders in individuals with pathogenic mtDNA variants [8]. Among these, DM stands out as the most prevalent, with an overall prevalence rate of 16.7% in those with the MERRF syndrome, which is higher than the reported 9.1% for diagnosed DM in the US population. Other endocrine disorders (such as hypogonadism and growth hormone deficiency), while rarer, have also been associated with mitochondrial dysfunction, including thyroid disease, hypoparathyroidism, and adrenal insufficiency [8].

Individuals with mitochondrial-related diabetes mellitus can have variable clinical presentations and can lead to either type 1 or type 2 DM. The disease may present itself insidiously, clinically similar to more typical age- and weight-related type 2 diabetes mellitus with both insulin resistance (due to mitochondrial dysfunction) and inadequate pancreatic beta-cell insulin secretion (leading to progressive β cell loss) [9]. These patients with mitochondrial diabetes mellitus exhibit an increased risk of progressing to require insulin treatment because glucose-stimulated insulin secretion is impaired. As reported recently, these patients require substantial amounts of bolus insulin (compared with basal insulin), which may also increase the risk of hypoglycemia in this context [10]. It is essential to exercise caution with certain medications, specifically metformin and thiazolidinediones, which should ideally be avoided due to their inhibitory effects on mitochondrial complex I, which may exacerbate lactic acidosis [6]. In our case, the patient exhibited high hyperlactacidemia while using metformin, which is consistent with the literature. Management of mitochondrial DM requires expertise and should always consider patients’ needs and preferences. In this particular case, the high burden of disease made us choose a simpler insulin regimen instead of a basal-bolus approach.

End-organ involvement in mitochondrial diseases also requires vigilant monitoring. Given the elevated risk of renal involvement, strict control of hypertension is recommended. If hypertension is present, the early use of either angiotensin-converting enzyme (ACE) inhibitors or angiotensin II receptor (ARB) blockers is highly recommended [6]. 

The typical onset age for MERRF syndrome is reportedly around 35 years old, with more severe forms often seen in those developing it during childhood compared to adulthood. Disease progression varies, even within the same family [11]. The patient described here developed neurological symptoms at 50 years old, slightly atypical for the condition. However, these neurological manifestations closely resemble classical MERRF symptoms, confirming the diagnostic spectrum [12]. The identification of the m.8344A>G variant in multiple family members, each displaying varying disease severity, mirrors the typical genetic inheritance pattern of MERRF syndrome. 

A noteworthy observation in this case is the initial presentation of endocrine dysfunction as the primary clinical feature preceding neurological symptoms, which, while uncommon, has been described before [6]. Specifically, our patient resembles a reported pediatric MELAS syndrome case that presented initially with adrenal insufficiency, and work-up revealed a m.8344A>G variant linked to MERRF syndrome, which is also present in our patient [13]. Although adrenal insufficiency is very rare in this context, it has been linked to mitochondrial diseases and, therefore, should be sought in the right clinical context. Due to the fact that adrenal insufficiency is a life-threatening but treatable condition, is it recommended that patients with mitochondrial diseases have their adrenal function assessed during critical illness [14].

Thyroid dysfunction, both hypothyroidism and, to a far lesser extent, hyperthyroidism, has been reported in patients with primary mitochondrial diseases and may coexist with other endocrine disorders [14]. In our patient, an episode of transient hyperthyroidism was present with negative antibodies. Whether this represents true thyroid dysfunction remains unknown. Regardless, annual TSH and free T4 are recommended for screening.

Nutritional support forms a cornerstone in the management of mitochondrial diseases, as certain dietary modifications and nutritional supplements have shown potential to ameliorate or stabilize disease manifestations. It is essential to note that while there is a growing body of anecdotal reports, robust scientific evidence from structured studies involving large populations is currently lacking. To our knowledge, no specific study has focused exclusively on MERRF syndrome and its relationship with nutritional support. Nonetheless, several supplements have been recommended to enhance mitochondrial function in the context of mitochondrial diseases. These include B-complex vitamins, coenzyme Q10, L-carnitine, alpha-lipoic acid, vitamin E, and creatine. In the absence of definitive clinical guidelines, the consideration of nutritional support should be individualized, considering the patient's unique needs and preferences [15]. In our patient, we opted to supplement with the coenzyme-Q and B vitamin complex due to its ease of access and low cost associated.

These observations underscore the need for heightened awareness and vigilance regarding endocrine function in patients with mitochondrial diseases, including MERRF syndrome.

## Conclusions

MERRF syndrome is a complex syndrome with several endocrine manifestations that have a major impact on the patient's quality of life and morbidity. A multidisciplinary approach involving endocrinologists, neurologists, geneticists, and other specialists is essential for providing comprehensive care to these patients. Moreover, our findings underscore the pressing need for targeted therapeutic strategies tailored to address both the neurological and endocrine aspects of MERRF syndrome, aiming to enhance patient outcomes and mitigate disease burden (which is particularly high in our patient).

The scarcity of specific case reports and literature solely dedicated to the endocrine manifestations of MERRF syndrome underscores the limited focus on this aspect within existing research. While our review aimed to shed light on the endocrine complexities of this syndrome, it's essential to acknowledge the lack of direct case reports or studies exclusively exploring these manifestations. The emphasis on registry data and indirect references within existing literature forms a crucial foundation for understanding the prevalence and spectrum of endocrine disorders in MERRF syndrome despite the paucity of dedicated studies. Moving forward, there's a pertinent need for more focused investigations and case reports that delve deeper into the intricate interplay between MERRF syndrome and its endocrine implications. Further research into the intricate interactions between mitochondrial dysfunction and endocrinopathies is warranted to improve both our understanding and management of these complex conditions.
